# Lipid levels and new-onset diabetes in a hypertensive population: the China Stroke Primary Prevention Trial

**DOI:** 10.1038/s41598-017-07355-w

**Published:** 2017-08-01

**Authors:** Leliang Li, Ping Li, Juan Yang, Xiao Huang, Huihui Bao, Chunyan Zhang, Yun Song, Min Zhao, Meng Ji, Yi Wang, Geng Qian, Genfu Tang, Shanqun Jiang, Qiang Dong, Yan Zhang, Jianping Li, Xiping Xu, Binyan Wang, Yong Huo, Xiaoshu Cheng

**Affiliations:** 1 Department of Cardiovascular Medicine, the Second Affiliated Hospital of Nanchang University, Nanchang of Jiangxi, China; 2 National Clinical Research Study Center for Kidney Disease, State Key Laboratory for Organ Failure Research, Renal Division, Nanfang Hospital, Southern Medical University, Guangzhou, China; 3 Shanghai Institute of Cardiovascular Diseases, Department of Cardiology, Zhongshan Hospital, Fudan University, Shanghai, China; 4 Department of Neurology, Huashan Hospital, Fudan University, Shanghai, China; 5 Department of Cardiology, General Hospital of PLA, Beijing, China; 6 Institute for Biomedicine, Anhui Medical University, Hefei, China; 7 School of Life Sciences, Anhui University, Hefei, China; 8 Department of Cardiology, Peking University First Hospital, Beijing, China

## Abstract

This study aimed to provide insights into the relationship between lipid levels and new-onset diabetes (NOD) in 14,864 Chinese hypertensive patients without diabetes (6056 men and 8808 women) aged 45–75 years from the China Stroke Primary Prevention Trial (CSPPT, led by Nanfang Hospital, Guangzhou, China). NOD (defined as fasting plasma glucose (FPG) ≥ 7.0 mmol/L at the end of study or self-reported physician diagnosis of diabetes or self-reported use of hypoglycemic agents during follow-up) was analyzed using multivariate analysis. Follow-up was censored on August 24, 2014. Among the 14,864 subjects, 1615 developed NOD (10.9%, men = 10.8% and women = 10.9%). Increased triglycerides (TG) [odds ratio (OR) = 1.18; 95% confidence interval (CI): 1.13–1.25, P < 0.001], TG/HDL (OR = 1.12; 95%CI: 1.08–1.17, P < 0.001), and decreased high density lipoprotein (HDL) (OR = 0.79; 95%CI: 0.67–0.93, P = 0.005) were associated with NOD, independently from age, gender, body mass index, clinical center, systolic blood pressure, diastolic blood pressure, FPG, smoking, and drinking. Compared to subjects with the methylenetetrahydrofolate reductase (MTHFR) 677 CC and TT genotypes, those with the CT genotype had a higher risk of NOD (OR = 1.54; 95%CI: 1.30–1.81, P for interaction = 0.044) in subjects with high TG. These results suggested that TG and TG/HDL were independent risk factors for NOD in this Chinese hypertensive population. HDL was a protective factor for NOD.

## Introduction

China, the most populous country in the world, is experiencing an increase in diabetes morbidity rates each year. A recent national cross-sectional survey showed that the overall prevalence of diabetes was estimated to be 11.6% in adult Chinese^1^. Previous studies found that impaired fasting glucose (IFG) or diabetes was positively associated with the risk of coronary heart disease (CHD)^2, 3^. In addition, diabetes is a major risk factor for ischemic heart disease (IHD) and stroke, which, in 2010, collectively contributed to an estimated global mortality of 12.9 million people^4^. In recent years, the consequences of unhealthy living habits have been shown to lead to a rapid increase in risk factors of diabetes and CHD, such as dyslipoproteinemia.

Several large randomized trials reported that baseline fasting blood glucose (FBG) levels are predictive for new-onset type 2 diabetes (NOD)^5, 6^. Indeed, Sattar *et al*.^7^ confirmed that high triglyceride levels (TG) (≥1.69 mmol/L) and low high-density lipoprotein (HDL) cholesterol (≤1.04 mmol/L for men and ≤1.29 mmol/L for women) are also predictors of diabetes. Tirosh *et al*.^8^ indicated that TG levels may identify people at an increased risk for diabetes, even in apparently healthy young men, independently of traditional risk factors, confirming that dyslipidemia may play an important role in identifying people at risk for NOD.

Most studies of the associations between reduced HDL-cholesterol or elevated TG levels and NOD risk are from European and American populations^7–9^ and there are very few studies on East Asian populations, especially hypertensive populations. In addition, the gender effect of higher HDL on NOD is unclear. Two studies indicated that higher HDL had a protective effect against NOD only among females^10, 11^. While Meisinger *et al*.^12^ showed that higher HDL was inversely associated with diabetes in both males and females.

Presently, there are no studies investigating the association between lipid levels and NOD in a Chinese hypertensive population. Therefore, the present study aimed to provide insights into the relationship between lipid levels and NOD in a Chinese hypertensive population, providing a better understanding of the relationship between HDL and NOD among males and females.

## Results

### Characteristics of the subjects

Follow-up was censored on August 24, 2014, and 14,864 subjects could be analyzed (71.8% of the original CSPPT study). Among them, 1615 (657 men and 958 women) developed NOD, for an incidence of 10.9%. The baseline characteristics of the participants are presented in Table 1. Individuals who developed NOD had significantly higher baseline fasting plasma glucose (FPG), body mass index (BMI), TG, and total cholesterol (TC), as well as lower HDL than those who did not develop diabetes (all P < 0.05). Females who developed NOD tended to be slightly older (59.8 vs. 59.1 years, P = 0.01) and to have higher baseline systolic blood pressure (SBP) (169.4 vs. 167.8 mmHg, P = 0.02) than non-diabetics, but these differences were not observed in males.

**Table 1:** Characteristics of the participants.

Variables	Males			Females		
	Non-diabetics	NOD	P	Non-diabetics	NOD	P
N	5399	657		7850	958	
Age, years	61.2 ± 7.4	61.4 ± 7.4	0.490	59.1 ± 7.3	59.8 ± 7.3	0.012
Angiotensin-converting enzyme inhibitor, n (%)	5399 (100.0)	657 (100.0)	—	7850 (100.0)	958 (100.0)	—
Treatment group, n (%)			0.319			0.905
Enalapril	2708 (50.2)	316 (48.1)		3941 (50.2)	479 (50.0)	
Enalapril-folic acid	2691 (49.8)	341 (51.9)		3909 (49.8)	479 (50.0)	
Clinical center, n (%)			0.420			0.146
Anqing	1569 (29.1)	181 (27.5)		1778 (22.6)	237 (24.7)	
Lianyungang	3830 (70.9)	476 (72.5)		6072 (77.4)	721 (75.3)	
Smoking status, n (%)			0.524			0.009
Never	1633 (30.3)	197 (30.0)		7534 (96.0)	899 (93.9)	
Ever	869 (16.1)	117 (17.8)		83 (1.1)	15 (1.6)	
Current	2896 (53.6)	343 (52.2)		228 (2.9)	43 (4.5)	
Alcohol, n (%)			0.041			0.263
Never	1869 (34.6)	211 (32.1)		7296 (93.0)	901 (94.1)	
Ever	679 (12.6)	105 (16.0)		212 (2.7)	26 (2.7)	
Current	2850 (52.8)	341 (51.9)		333 (4.2)	30 (3.1)	
BMI, kg/m^2^	24.0 ± 3.3	25.0 ± 3.7	<0.001	25.2 ± 3.7	26.5 ± 4.0	<0.001
SBP, mmHg	165.4 ± 20.3	166.2 ± 21.4	0.317	167.8 ± 20.1	169.4 ± 20.8	0.022
DBP, mmHg	95.5 ± 12.3	95.6 ± 12.5	0.807	93.3 ± 11.4	93.8 ± 11.3	0.254
TG, mmol/l	1.5 ± 0.9	1.8 ± 1.4	<0.001	1.7 ± 0.8	2.0 ± 3.2	<0.001
HDL, mmol/l	1.4 ± 0.4	1.3 ± 0.4	0.002	1.3 ± 0.3	1.3 ± 0.3	<0.001
TC, mmol/l	5.3 ± 1.1	5.4 ± 1.2	0.019	5.6 ± 1.2	5.6 ± 1.2	0.794
FPG, mmol/l	5.3 ± 0.7	5.7 ± 0.8	<0.001	5.4 ± 0.6	5.8 ± 0.7	<0.001
Crea, μmol/L	76.5 ± 16.9	74.3 ± 14.4	0.002	58.7 ± 11.9	57.6 ± 11.5	0.006
Folate, median (IQR), pg/ml	7.1 (0.7–49.1)	6.9 0.9–21.8)	0.460	8.5 (1.4–67.8)	8.1(2.7–38.7)	0.006
B12, median (IQR), μmol/L	370.3(39.6–2947.5)	374.2(92.7–1644.5)	0.363	377.3(9.9–4467.0)	380.8(69.3–1939.7)	0.147
Hcy, median (IQR), μmol/l	14.1 (3.0–142.3)	14.3 (5.0–119.4)	0.864	11.4 (3.5–113.5)	11.6 (5.1–52.6)	0.894
MTHFR C677T, n (%)			0.157			0.386
CC	1515 (28.1)	176 (26.8)		2141 (27.3)	260 (27.1)	
CT	2644 (49.0)	308 (46.9)		3901 (49.7)	459 (47.9)	
TT	1240 (23.0)	173 (26.3)		1808 (23.0)	239 (24.9)	

Continuous variables are presented as mean ± SD or median (IQR), while categorical variables are presented as n (%).

Abbreviations: SBP = systolic blood pressure, DBP = diastolic blood pressure, TG = triglycerides, HDL = high-density lipoprotein, TC = total cholesterol, FPG = fasting plasma glucose, HCY = homocysteine, BMI = body mass index, NOD = new-onset diabetes; MTHFR = methylenetetrahydrofolate reductase.

### Association between lipid levels and NOD

Both continuous and categorical TG, TG/HDL, and HDL levels were significantly associated with incident diabetes (all P < 0.001). They were independent predictors of diabetes after adjusting for other covariables. The incidence of NOD was significantly higher in subjects with high TG levels compared to those with normal TG levels (13.9% vs. 9.2%, P < 0.001) and with high TG/HDL ratio compared to those with normal (17.2% vs. 10.4%, P < 0.001), while significantly lower when HDL levels were elevated (9.8% vs. 12.8%, P < 0.001) (Table 2). Compared to subjects with normal TG levels (<1.7 mmol/L), those with high TG levels (≥1.7 mmol/L) had a higher risk of NOD [odds ratio (OR) = 1.35; 95%CI confidence interval (95%CI): 1.20–1.51). Similarly, high TG/HDL ratio (≥2.8) had a higher risk of NOD (OR = 1.46; 95%CI: 1.22–1.75), while high HDL levels [≥1.0 (males)/1.3 (females) mmol/L] were negatively associated with NOD (OR = 0.82; 95%CI: 0.72–0.92) compared to subjects with low HDL levels [<1.0 (males)/1.3 (females) mmol/L]. TC was not associated with NOD (P = 0.10) when only adjusted for age, but after adjusting for other covariables, TC was negatively associated with NOD (P = 0.03). This suggests that TC alone is a protective factor of NOD, but that its effect is easily influenced by other factors.

**Table 2:** Odds ratios of lipid levels for NOD by logistic regression.

Variables	Events (%)/n	Age-adjusted		Multivariate adjusted *	
		OR (95%CI)	P value	OR (95%CI)	P value
**TG**, **mmol/l**					
Continuous	1615 (10.9)/14,864	1.29 (1.23, 1.35)	<0.001	1.18 (1.13, 1.25)	<0.001
Categorical					
<1.7	901 (9.2)/9743	ref		ref	
>1.7	714 (13.9)/5121	1.60 (1.44, 1.78)	<0.001	1.35 (1.20, 1.51)	<0.001
**HDL**, **mmol/l**					
Continuous	1615 (10.9)/14864	0.61 (0.53, 0.71)	<0.001	0.79 (0.67, 0.93)	0.005
Categorical					
<1.0 (males)/1.3 (females)	678 (12.8)/5302	ref		ref	
≥1.0 (males)/1.3 (females)	937 (9.8)/9562	0.73 (0.66, 0.81)	<0.001	0.82 (0.72, 0.92)	<0.001
**TC**, **mmol/l**					
Continuous	1615 (10.9)/14,864	1.04 (0.99 1.09)	0.100	0.95 (0.90, 1.00)	0.033
Categorical					
<5.2	680 (10.7)/6342	ref		ref	
>5.2	935 (11.0)/8522	1.03 (0.93, 1.14)	0.602	0.87 (0.78, 0.98)	0.017
**TG/HDL**					
Continuous	1615 (10.9)/14,864	1.21 (1.17, 1.26)	<0.001	1.12 (1.08, 1.17)	<0.001
Categories					
<2.8	1437 (10.4)/13,832	ref		ref	
≥2.8	178 (17.2)/1032	1.82 (1.53, 2.16)	<0.001	1.46 (1.22, 1.75)	<0.001

Abbreviations: TG = triglycerides, HDL = high-density lipoprotein, TC = total cholesterol, NOD = new-onset diabetes.

*Adjusted for age, gender, clinical center, SBP, DBP, smoking and drinking status, baseline FPG, and BMI.

### Subgroup analyses of factors influencing the association between TG and NOD

We further explored the role of other covariables on the association between lipid profiles and NOD. Table 3 shows the results of a subgroup analysis assessing the risk of NOD associated with TG. TG was positively associated with NOD, and it was more significant in patients from the Anqing district (Anqing: OR = 1.27, 95%CI: 1.13–1.43; Lianyungang: OR = 1.16, 95%CI: 1.10–1.23, P for interaction = 0.026). Compared to subjects with the MTHFR 677 CC and TT genotypes, those with the CT genotype had a higher risk of NOD (OR = 1.54, 95%CI, 1.30–1.81, P for interaction = 0.044) in subjects with high TG levels. The effect of TG on NOD showed no difference in the following subgroups: age, gender, treatment group (enalapril vs. enalapril-folic acid), smoking, drinking, BMI, SBP, DBP, folic acid, and homocysteine (Hcy) levels (all P for interaction >0.10) (Table 3).

**Table 3:** Subgroup analysis of NOD risk associated with TG according to covariates by logistic regression.

Subgroups	Continuous				Categorical				P for interaction between the two factors
	Events, n (%)/n	OR (95%CI)	P value	P for interaction between the two factors	Normal	Abnormal	OR (95%CI)	P value	
					Events, n (%)/N	Events, n (%)/N			
Age				0.792					0.533
<60	777 (10.4)/7648	1.18 (1.11, 1.26)	<0.001		419 (8.9)/4718	358 (13.0)/2750	1.30 (1.10, 1.52)	0.002	
≥60	838 (11.3)/7396	1.20 (1.10, 1.30)	<0.001		482 (9.6)/5025	356 (15.0)/2731	1.41 (1.20, 1.65)	<0.001	
Center*				0.026					0.028
Anqing	418 (11.1)/3765	1.27 (1.13, 1.43)	<0.001		253 (9.2)/2763	165 (16.5)/1002	1.57 (1.24, 2.00)	<0.001	
Lianyungang	1197 (10.8)/11,099	1.16 (1.10, 1.23)	<0.001		648 (9.3)/6980	549 (13.3)/4119	1.28 (1.12, 1.45)	<0.001	
Gender				0.415					0.896
Males	657 (10.8)/6056	1.17 (1.09, 1.25)	<0.001		417 (9.4)/4432	240 (14.8)/1624	1.38 (1.14, 1.66)	<0.001	
Females	958 (10.9)/8808	1.21 (1.12, 1.30)	<0.001		484 (9.1)/5311	474 (13.6)/3497	1.33(1.15, 1.54)	<0.001	
Treatment group				0.767					0.974
Enalapril	795 (10.7)/7444	1.18 (1.09, 1.27)	<0.001		442 (9.1)/4855	353 (13.6)/2589	1.30 (1.10, 1.52)	0.002	
Enalapril-folic acid	820 (11.1)/7420	1.20 (1.12, 1.29)	<0.001		459 (9.4)/4888	361 (14.3)/2532	1.41 (1.20, 1.66)	<0.001	
Smoking status				0.352					0.223
Never	1096 (10.7)/10,263	1.16 (1.09, 1.24)	<0.001		587 (9.2)/6400	509 (13.2)/3863	1.27 (1.11, 1.46)	<0.001	
Ever	132 (12.2)/1084	1.14 (0.92, 1.41)	0.234		78 (10.2)/762	54 (16.8)/322	1.46 (0.97, 2.22)	0.073	
Current	386 (11.0)/3510	1.23 (1.12, 1.35)	<0.001		235 (9.1)/2577	151 (16.2)/933	1.53 (1.20, 1.96)	<0.001	
Alcohol				0.430					0.957
Never	1112 (10.8)/10,277	1.21 (1.13, 1.29)	<0.001		582 (9.0)/6435	530 (13.8)/3842	1.35 (1.18, 1.54)	<0.001	
Ever	131 (12.8)/1022	1.32 (1.05, 1.65)	0.016		82 (11.5)/634	49 (16.0)/306	1.35 (0.881, 2.07)	0.168	
Current	371 (10.4)/3554	1.14 (1.05, 1.24)	0.002		236 (9.1)/2584	135 (13.9)/970	1.34 (1.04, 1.72)	0.022	
BMI				0.486					
<24	523 (8.3)/6283	1.12 (1.02, 1.22)	0.015		393 (7.9)/4947	130 (9.7)/1336	1.16 (0.95, 1.43)	0.139	0.345
≥24, <28	643 (11.1)/5786	1.14 (1.07, 1.21)	<0.001		339 (9.8)/3468	304 (13.1)/2318	1.28 (1.09, 1.49)	0.002	
≥28	448 (16.1)/2790	1.04 (1.02, 1.05)	<0.001		168 (12.7)/1323	280 (19.1)/1467	1.36 (1.12, 1.65)	0.002	
SBP, mmHg				0.304					0.930
<140	90 (10.6)/853	1.36 (1.08, 1.72)	0.009		48 (8.5)/562	42 (14.4)/291	1.43 (0.87, 2.34)	0.160	
≥140, <160	495 (10.4)/4759	1.19 (1.08, 1.31)	<0.001		276 (8.7)/3180	219 (13.9)/1579	1.34 (1.09, 1.65)	0.005	
≥160, <180	595 (10.5)/5691	1.12 (1.03, 1.22)	0.011		334 (8.9)/3739	261 (13.4)/1952	1.31 (1.09, 1.58)	0.004	
≥180	435 (12.2)/3561	1.21 (1.11, 1.33)	<0.001		243 (10.7)/2262	192 (14.8)/1299	1.36 (1.09, 1.69)	0.006	
DBP, mmHg				0.884					0.344
<90	531 (10.9/4882)	1.19 (1.07, 1.32)	0.001		309 (9.2)/3360	222 (14.6)/1522	1.40 (1.15, 1.72)	<0.001	
≥90, <100	551 (10.8)/5125	1.17 (1.07, 1.28)	<0.001		308 (9.2)/3336	243 (13.6)/1789	1.33 (1.09, 1.61)	0.004	
≥100, <110	368 (10.9)/3390	1.16 (1.04, 1.29)	0.007		204 (9.5)/2142	164 (13.1)/1248	1.19 (0.94, 1.50)	0.148	
≥110	165 (11.2)/1467	1.26 (1.13, 1.42)	<0.001		80 (8.8)/905	85 (15.1)/562	1.68 (1.18, 2.39)	0.004	
Folic acid, ng/ml				0.672					0.104
<8.1	860 (11.5)/7510	1.17 (1.09, 1.25)	<0.001		461 (10.0)/4623	399 (13.8)/2887	1.21 (1.04, 1.41)	0.016	
≥8.1	746 (10.3)/7233	1.20 (1.11, 1.30)	<0.001		434 (8.6)/5044	312 (14.3)/2189	1.50 (1.27, 1.78)	<0.001	
Hcy, μmol/l				0.565					0.418
<10	305 (10.4)/2944	1.14 (1.02, 1.27)	0.018		160 (8.4)/1896	145 (13.8)/1048	1.42 (1.10, 1.84)	0.008	
≥10	1310 (11.0)/11,914	1.20 (1.13, 1.27)	<0.001		741 (9.4)/7844	569 (14.0)/4070	1.33 (1.17, 1.50)	<0.001	
MTHFR C677T,				0.064					0.044
CC	436 (10.7)/4092	1.13 (1.03, 1.24)	0.008		251 (9.2)/2762	185 (13.5)/1366	1.28 (1.03, 1.60)	0.029	
CT	767 (10.5)/7312	1.27 (1.17, 1.37)	<0.001		408 (8.5)/4796	359 (14.3)/2516	1.54 (1.30, 1.81)	<0.001	
TT	412 (11.9)/3460	1.11 (1.00, 1.23)	0.050		242 (10.9)/2221	170 (13.7)/1239	1.11 (0.885, 1.39)	0.369	

Abbreviations: SBP = systolic blood pressure, DBP = diastolic blood pressure, TG = triglycerides, HDL = high-density lipoprotein, TC = total cholesterol, HCY = homocysteine, NOD = new-onset diabetes. MTHFR = methylenetetrahydrofolate reductase.

Adjusted for age, gender, clinical center, SBP, DBP, smoking and drinking status, baseline FPG, and BMI, if not stratified. Only TG levels were analyzed because of colinearity with TC and HDL.

### Subgroup analyses of factors influencing the association between TC and NOD

We then analyzed the relationship between TC and NOD. The data suggest that TC was protective in the enalapril-folic acid group (enalapril-folic acid: OR = 0.91, 95%CI: 0.85–0.98; enalapril: OR = 0.98, 95%CI: 0.92–1.05, P for interaction = 0.034). Compared to subjects who ever smoked or currently smoke, those who never smoked had a significant protective effect of NOD (OR = 0.92, 95%CI: 0.87–0.98, P for interaction = 0.036). TC appeared to be negatively associated with NOD, but the stratified analyses show that in each subgroup presented above, TC is not associated with NOD (P for interaction >0.05). In addition, the effect of TC on NOD showed no differences within the subgroups of age, clinical center, gender, alcohol, BMI, SBP, DBP, MTHFR C677T, folic acid, and Hcy (all P for interaction >0.05) (Table 4).

**Table 4:** Subgroup analysis of NOD risk associated with TC according to important covariates by logistic regression.

Subgroups	Continuous				Categorical				P for interaction between the two factors
	Events, n (%)/N	OR (95%CI)	P value	P for interaction between the two factors	Normal	Abnormal	OR (95%CI)	P value	
					Events, n (%)/N	Events, n (%)/N			
Age				0.517					0.47
<60	777 (10.4)/7648	0.97 (0.90, 1.04)	0.394		322 (10.4)/3087	455 (10.4)/4381	0.85 (0.72, 1.00)	0.044	
≥60	838 (11.3)/7396	0.93 (0.86, 0.99)	0.033		358 (11.0)/3255	480 (11.6)/4141	0.90 (0.76, 1.05)	0.173	
Center*				0.938					0.458
Anqing	418 (11.1)/3765	0.93 (0.83, 1.04)	0.195		259 (10.8)/2393	159 (11.6)/1372	0.90 (0.72, 1.12)	0.345	
Lianyungang	1197 (10.8)/11,099	0.95 (0.90, 1.00)	0.066		421 (10.7)/3949	776 (10.9)/7150	0.85(0.74, 0.97)	0.015	
Gender				0.079					0.199
Males	657 (10.8)/6056	1.00 (0.93, 1.09)	0.918		308 (10.4)/2953	349 (11.2)/3103	0.94(0.79, 1.12)	0.508	
Females	958 (10.9)/8808	0.91 (0.86, 0.97)	0.005		372 (11.0)/3389	586 (10.8)/5419	0.82 (0.70, 0.95)	0.008	
Treatment-group				0.034					0.253
Enalapril	795 (10.7)/7444	0.98 (0.92, 1.05)	0.633		329 (10.3)/3196	466 (11.0)/4248	0.90(0.76, 1.05)	0.184	
Enalapril-folic acid	820 (11.1)/7420	0.91 (0.85, 0.98)	0.011		351 (11.2)3146	469 (11.0)/4274	0.85 (0.72, 0.99)	0.039	
Smoking status				0.036					0.054
Never	1096 (10.7)/10,263	0.92 (0.87, 0.98)	0.008		458 (10.9)/4210	638 (10.5)/6053	0.82(0.72, 0.94)	0.006	
Ever	132 (12.2)/1084	0.90 (0.75, 1.08)	0.259		64 (12.4)/517	68 (12.0)/567	0.77 (0.51, 1.14)	0.193	
Current	386 (11.0)/3510	0.99 (0.89, 1.10)	0.877		157 (9.7)/1612	229 (12.1)/1898	1.05 (0.82, 1.33)	0.714	
Alcohol				0.088					0.169
Never	1112 (10.8)/10,277	0.95 (0.89, 1.01)	0.073		463 (10.7)/4329	649 (10.9)/5948	0.86 (0.75, 0.99)	0.032	
Ever	131 (12.8)/1022	0.81 (0.67, 0.99)	0.034		76 (13.8)/550	55 (11.7)/472	0.68 (0.45, 1.02)	0.064	
Current	371 (10.4)/3554	0.98 (0.89, 1.09)	0.769		140 (9.6)/1459	231 (11.0)/2095	0.97 (0.76, 1.23)	0.794	
BMI				0.436					0.523
<24	523 (8.3)/6283	0.85 (0.78, 0.93)	<0.001		260 (8.4)/3093	263 (8.2)/3190	0.71 (0.59, 0.86)	<0.001	
≥24, <28	643 (11.1)/5786	0.79 (0.73, 0.85)	<0.001		262 (11.7)/2245	381 (10.8)/3541	0.64 (0.54, 0.76)	<0.001	
≥28	448 (16.1)/2790	0.85(0.78, 0.92)	<0.001		158 (15.7)/1004	290 (16.2)/1786	0.69 (0.56, 0.84)	<0.001	
SBP, mmHg				0.990					0.789
<140	90 (10.6)/853	1.00 (0.80, 1.25)	0.971		46 (10.7)/428	44 (10.4)/425	0.86 (0.53, 1.40)	0.547	
≥140, <160	495 (10.4)/4759	0.95 (0.87, 1.04)	0.274		232 (10.5)/2218	263 (10.4)/2541	0.82 (0.67, 1.00)	0.055	
≥160, <180	595 (10.5)/5691	0.94 (0.87, 1.03)	0.170		251 (10.6)/2379	344 (10.4)/3312	0.85 (0.70, 1.03)	0.091	
≥180	435 (12.2)/3561	0.94 (0.86, 1.04)	0.225		151 (11.5)/1317	284 (12.7)/2244	0.96 (0.77, 1.20)	0.731	
DBP, mmHg				0.756					0.910
<90	531 (10.9/4882)	0.95 (0.87, 1.04)	0.283		254 (10.9)/2340	277 (10.9)/2542	0.84 (0.69, 1.02)	0.085	
≥90, <100	551 (10.8)/5125	0.93 (0.85, 1.01)	0.075		231 (10.8)/2145	320 (10.7)/2980	0.87(0.72, 1.06)	0.166	
≥100, <110	368 (10.9)/3390	0.93 (0.84, 1.04)	0.201		142 (10.7)/1328	226 (11.0)/2062	0.84 (0.66, 1.07)	0.162	
≥110	165 (11.2)/1467	1.01 (0.87, 1.18)	0.854		53 (10.0)/529	112 (11.9)/938	1.01 (0.70, 1.47)	0.956	
Folic acid, ng/ml				0.647					0.355
<8.1	860 (11.5)/7510	0.96 (0.89, 1.02)	0.201		339 (11.1)/3064	521 (11.7)/4446	0.91 (0.78, 1.07)	0.257	
>8.1	746 (10.3)/7233	0.94 (0.87, 1.01)	0.097		336 (10.4)/3216	410 (10.2)/4017	0.83 (0.70, 0.98)	0.031	
Hcy, μmol/l				0.66					0.967
<10	305 (10.4)/2944	0.96 (0.86, 1.07)	0.474		131 (10.0)/1312	174 (10.7)/1632	0.86 (0.67, 1.12)	0.271	
>10	1310 (11.0)/11,914	0.94 (0.89, 1.00)	0.033		549 (10.9)/5028	761 (11.1)/6886	0.87 (0.76, 0.99)	0.029	
MTHFR C677T				0.927					0.507
CC	436 (10.7)/4092	0.95 (0.86, 1.05)	0.295		202 (10.7)/1885	234 (10.6)/2207	0.84 (0.67, 1.05)	0.119	
CT	767 (10.5)/7312	0.94 (0.87, 1.01)	0.085		330 (10.7)/3098	437 (10.4)/4214	0.84 (0.71, 0.98)	0.032	
TT	412 (11.9)/3460	0.96 (0.87, 1.06)	0.440		148 (10.9)/1359	264 (12.6)/2101	0.97 (0.77, 1.22)	0.777	

SBP = systolic blood pressure, DBP = diastolic blood pressure, TG = triglycerides, HDL = high-density lipoprotein, TC = total cholesterol, HCY = homocysteine, NOD = new-onset diabetes. MTHFR = methylenetetrahydrofolate reductase.

*Clinical center represents two rural areas in Anhui and Jiangsu provinces in China.

Adjusted for age, gender, clinical center, SBP, DBP, smoking and drinking status, baseline FPG, and BMI, if not stratified. Only TG levels were analyzed because of colinearity with TC and HDL.

### Subgroup analyses of factors influencing the association between HDL and NOD

HDL was negatively associated with NOD, and was more significant for patients from the Anqing district (Anqing: OR = 0.42, 95%CI: 0.29–0.61; Lianyungang: OR = 0.94; 95%CI: 0.78–1.13, P for interaction <0.001). This was also true for individuals with high folic acid levels at baseline (≥8.1 ng/ml: OR = 0.65, 95%CI: 0.51–0.83; <8.1 ng/ml: OR = 0.95, 95%CI: 0.76–1.19, P for interaction = 0.027). In addition, stratified analyses were performed by MTHFR C677T genotypes (CC, CT, and TT), age, gender, treatment group (enalapril vs. enalapril-folic acid), smoking, drinking, BMI, SBP, DBP, and Hcy level, but there were no significant interactions in any of the subgroups (all P > 0.05), including gender (P = 0.199) (Table 5).

**Table 5:** Subgroup analysis of NOD risk associated with HDL according to important covariates by logistic regression.

Subgroups	Continuous				Categorical				P for interaction between the two factors
	Events, n (%)/N	OR (95%CI)	P value	P for interaction between the two factors	Normal	Abnormal	OR (95%CI)	P value	
					Events, n (%)/N	Events, n (%)/N			
Age				0.293					0.321
<60	777 (10.4)/7648	0.87 (0.69, 1.11)	0.262		340 (11.9)/2862	437 (9.5)/4606	0.86 (0.72, 1.02)	0.083	
≥60	838 (11.3)/7396	0.71 (0.56, 0.89)	0.004		338 (13.9)/2440	500 (10.1)/4956	0.77 (0.65, 0.92)	0.003	
Center*				<0.001					0.028
Anqing	418 (11.1)/3765	0.42 (0.29, 0.61)	<0.001		179 (15.3)/1173	239 (9.2)/2592	0.61 (0.47, 0.79)	<0.001	
Lianyungang	1197 (10.8)/11,099	0.94 (0.78, 1.13)	0.492		499 (12.1)/4129	698 (10.0)/6970	0.89 (0.77, 1.02)	0.098	
Gender				0.199					0.262
Males	657 (10.8)/6056	0.88 (0.67, 1.13)	0.311		115 (12.9)/892	542 (10.5)/5164	0.92 (0.73, 1.16)	0.498	
Females	958 (10.9)/8808	0.73 (0.58, 0.91)	0.006		563 (12.8)/4410	395 (9.0)/4398	0.79 (0.68, 0.91)	0.001	
Treatment group				0.922					0.194
Enalapril	795 (10.7)/7444	0.79 (0.63, 1.01)	0.057		348 (13.1)/2665	447 (9.4)/4779	0.78 (0.65, 0.92)	0.004	
Enalapril-folic acid	820 (11.1)/7420	0.77 (0.61, 0.97)	0.027		330 (12.5)/2637	490 (10.2)/4783	0.85 (0.72, 1.01)	0.063	
Smoking status				0.598					0.458
Never	1096 (10.7)/10263	0.76(0.62, 0.94)	0.010		569 (12.5)/4548	527 (9.2)/5715	0.80 (0.70, 0.92)	0.002	
Ever	132 (12.2)/1084	0.62 (0.35, 1.12)	0.111		37 (16.9)/219	95 (11.0)/865	0.63 (0.39, 1.00)	0.048	
Current	386 (11.0)/3510	0.94 (0.68, 1.28)	0.682		71 (13.3)/532	315 (10.6)/2978	1.01 (0.74, 1.38)	0.954	
Alcohol				0.356					0.030
Never	1112 (10.8)/10,277	0.77 (0.63, 0.95)	0.016		582 (12.7)/4589	530 (9.3)/5688	0.83 (0.73, 0.95)	0.008	
Ever	131 (12.8)/1022	0.52 (0.27, 0.99)	0.048		52 (18.3)/284	79 (10.7)/738	0.47 (0.31, 0.72)	<0.001	
Current	371 (10.4)/3554	0.90 (0.66, 1.21)	0.477		43 (10.1)/424	328 (10.5)/3130	1.08 (0.75, 1.55)	0.686	
BMI				0.242					0.652
<24	523 (8.3)/6283	0.72 (0.56, 0.92)	0.008		129 (8.7)/1491	394 (8.2)/4792	0.74(0.59, 0.92)	0.007	
≥24, <28	643 (11.1)/5786	0.57 (0.45, 0.74)	<0.001		293 (12.4)/2368	350 (10.2)/3418	0.67 (0.56, 0.79)	<0.001	
≥28	448 (16.1)/2790	0.49 (0.35, 0.69)	<0.001		256 (17.7)/1443	192 (14.3)/1347	0.65 (0.53, 0.80)	<0.001	
SBP, mmHg				0.524					0.800
<140	90 (10.6)/853	0.96(0.45, 2.05)	0.913		41 (13.0)/316	49 (9.1)/537	0.73 (0.43, 1.23)	0.239	
≥140, <160	495 (10.4)/4759	0.72 (0.53, 0.98)	0.035		214 (12.4)/1719	281 (9.2)/3040	0.83 (0.66, 1.03)	0.089	
≥160, <180	595 (10.5)/5691	0.80 (0.61, 1.05)	0.103		248 (12.4)/2004	347 (9.4)/3687	0.82 (0.67, 1.00)	0.049	
≥180	435 (12.2)/3561	0.83 (0.61, 1.12)	0.223		175 (13.9)/1263	260 (11.3)/2298	0.82 (0.65, 1.04)	0.100	
DBP, mmHg				0.182					0.430
<90	531 (10.9/4882)	0.67 (0.50, 0.90)	0.008		240 (13.3)/1811	291 (9.5)/3071	0.76 (0.62, 0.94)	0.010	
≥90, <100	551 (10.8)/5125	0.84(0.63, 1.11)	0.224		228 (12.3)/1847	323 (9.9)/3278	0.81 (0.66, 0.99)	0.044	
≥100, <110	368 (10.9)/3390	0.97 (0.70, 1.35)	0.863		143 (12.3)/1166	225 (10.1)/2224	0.95 (0.74, 1.24)	0.721	
≥110	165 (11.2)/1467	0.57 (0.33, 0.99)	0.047		67 (14.0)/478	98 (9.9)/989	0.75 (0.51, 1.11)	0.146	
Folic acid, ng/ml				0.027					0.498
<8.1	860 (11.5)/7510	0.95 (0.76, 1.19)	0.678		370 (13.3)/2784	490 (10.4)/4726	0.88 (0.74, 1.04)	0.135	
≥8.1	746 (10.3)/7233	0.65 (0.51, 0.83)	<0.001		305 (12.3)/2472	441 (9.3)/4761	0.76 (0.64, 0.91)	0.003	
Hcy, μmol/l				0.457					0.504
<10	305 (10.4)/2944	0.73 (0.50, 1.07)	0.110		156 (12.4)/1262	149 (8.9)/1682	0.76 (0.58, 0.99)	0.043	
≥10	1310 (11.0)/11,914	0.80 (0.66, 0.96)	0.016		522 (12.9)/4036	788 (10.0)/7878	0.83 (0.72, 0.95)	0.007	
MTHFR C677T				0.055					0.193
CC	436 (10.7)/4092	0.62(0.45, 0.86)	0.004		179 (12.6)/1417	257 (9.6)/2675	0.82 (0.64, 1.03)	0.092	
CT	767 (10.5)/7312	0.78 (0.61, 0.99)	0.043		338 (12.8)/2644	429 (9.2)/4668	0.76 (0.64, 0.91)	0.002	
TT	412 (11.9)/3460	1.01 (0.74, 1.39)	0.936		161 (13.0)/1241	251 (11.3)/2219	0.93 (0.73, 1.19)	0.572	

SBP = systolic blood pressure, DBP = diastolic blood pressure, TG = triglycerides, HDL = high-density lipoprotein, TC = total cholesterol, HCY = homocysteine, NOD = new-onset diabetes, MTHFR = methylenetetrahydrofolate reductase.

*Clinical center represents two rural areas in Anhui and Jiangsu provinces in China.

Adjusted for age, gender, clinical center, SBP, DBP, smoking and drinking status, baseline FPG, and BMI, if not stratified.

Only TG levels were analyzed because of colinearity with TC and HDL.

### Subgroup analyses of factors influencing the association between TG/HDL and NOD

We further explored the role of other covariables on the association between lipid profiles and NOD. Table 6 shows the results of a subgroup analysis assessing the risk of NOD with TG/HDL. TG/HDL was positively associated with NOD, and it was more significant in patients from the Anqing district (Anqing: OR = 1.18, 95%CI: 1.09–1.28; Lianyungang: OR = 1.10, 95%CI: 1.05–1.16, P for interaction = 0.028). The effect of TG/HDL on NOD showed no difference in the following subgroups: age, gender, treatment group (enalapril vs. enalapril-folic acid), smoking, drinking, BMI, SBP, DBP, folic acid, MTHFR C677T genotypes and Hcy (homocysteine) levels (all P for interaction >0.10) (Table 6).

**Table 6:** Subgroup analysis of NOD risk associated with TG/HDL according to important covariates by logistic regression.

Subgroups	Continuous				Categories				P for interaction
	Events, n (%)/N	OR (95%CI)	P value	P for interaction	Normal	Abnormal	OR (95%CI)	P value	
					Events, n (%)/N	Events, n (%)/N			
Age				0.527					0.833
<60	777 (10.4)/7648	1.12 (1.06, 1.18)	<0.001		682 (9.9)/6889	95 (16.4)/579	1.45 (1.13, 1.86)	0.004	
≥60	838 (11.3)/7396	1.15 (1.07, 1.23)	<0.001		755 (10.9)/ 6943	83 (18.3)/453	1.47 (1.13, 1.92)	0.004	
Center*				0.028					0.007
Anqing	418 (11.1)/3765	1.18 (1.09, 1.28)	<0.001		370 (10.4)/3560	48 (23.4)/205	2.19 (1.50, 3.18)	<0.001	
Lianyungang	1197 (10.8)/11,099	1.10 (1.05, 1.16)	<0.001		1067 (10.4)/10272	130 (15.7)/827	1.30 (1.06, 1.61)	0.013	
Gender				0.498					0.553
Males	657 (10.8)/6056	1.11 (1.04, 1.18)	0.001		581 (10.3)/5653	76 (18.9)/403	1.58 (1.19, 2.10)	0.002	
Females	958 (10.9)/8808	1.14 (1.07, 1.21)	<0.001		856 (10.5)/8179	102 (16.2)/629	1.46 (1.22, 1.75)	<0.001	
Group				0.790					0.890
Enalapril	795 (10.7)/7444	1.12 (1.05, 1.20)	<0.001		707 (10.2)/6937	88 (17.4)/507	1.44 (1.11, 1.87)	0.006	
Enalapril-folic acid	820 (11.1)/7420	1.14 (1.07, 1.21)	<0.001		730 (10.6)/6895	90 (17.1)/525	1.50 (1.16, 1.93)	0.002	
Smoke status				0.544					0.318
Never	1096 (10.7)/10,263	1.11 (1.05, 1.17)	<0.001		979 (10.3)/9530	117 (16.0)/733	1.39 (1.11, 1.73)	0.003	
Ever	132 (12.2)/1084	1.13 (0.95, 1.35)	0.163		118 (11.8)/1001	14 (16.9)/83	1.26 (0.65, 2.42)	0.492	
Current	386 (11.0)/3510	1.16 (1.07, 1.26)	<0.001		339 (10.3)/3294	47 (21.8)/216	1.79 (1.23, 2.60)	0.002	
Drink status				0.374					0.942
Never	1112 (10.8)/10,277	1.14 (1.08, 1.20)	<0.001		987 (10.4)/9528	125 (16.7)/749	1.44 (1.16, 1.78)	0.001	
Ever	131 (12.8)/1022	1.25 (1.04, 1.50)	0.015		115 (12.2)/945	16 (20.8)/77	1.51 (0.79, 2.86)	0.216	
Current	371 (10.4)/3554	1.09 (1.01, 1.17)	0.023		334 (10.0) 3348	37 (18.0)/206	1.57 (1.05, 2.35)	0.028	
BMI				0.743					0.750
<24	523 (8.3)/6283	1.12 (1.02, 1.23)	0.017		505 (8.3)/ 6102	18 (9.9)/ 181	1.29 (0.78, 2.14)	0.320	
≥24, <28	643 (11.1)/5786	1.14 (1.07, 1.22)	<0.001		566 (10.6)/ 5322	77 (16.6)/ 464	1.55 (1.18, 2.03)	0.001	
≥28	448 (16.1)/2790	1.14 (1.06, 1.23)	<0.001		365 (15.2)/ 2403	83 (21.4)/ 387	1.56 (1.17, 2.08)	0.003	
SBP, mmHg				0.220					0.765
<140	90 (10.6)/853	1.24 (1.02, 1.50)	0.028		77 (9.7)/ 794	13 (22.0)/ 59	1.99 (0.94, 4.25)	0.074	
≥140, <160	495 (10.4)/4759	1.14 (1.06, 1.24)	<0.001		440 (9.9)/ 4433	55 (16.9)/326	1.36 (0.98, 1.90)	0.068	
≥160, <180	595 (10.5)/5691	1.07 (0.99, 1.14)	0.072		535 (10.1)/ 5311	60 (15.8)/380	1.36 (1.00, 1.85)	0.048	
≥180	435 (12.2)/3561	1.16 (1.07, 1.26)	0.001		385 (11.7)/ 3294	50 (18.7)/267	1.62 (1.15, 2.28)	0.006	
DBP, mmHg				0.934					0.268
<90	531 (10.9/4882	1.14 (1.05, 1.24)	0.001		481 (10.5)/ 4583	50 (16.7)/ 299	1.35 (0.96, 1.89)	0.090	
≥90, <100	551 (10.8)/5125	1.11 (1.03, 1.20)	0.006		498 (10.4)/ 4782	53 (15.5)/343	1.25 (0.90, 1.72)	0.185	
≥100, <110	368 (10.9)/3390	1.11 (1.01, 1.22)	0.023		320 (10.2)/ 3123	48 (18.0)/267	1.58 (1.11, 2.26)	0.011	
≥110	165 (11.2)/1467	1.16 (1.04, 1.28)	0.005		138 (10.3)/1344	27 (22.0)/123	2.39 (1.45, 3.95)	0.001	
Folic acid, ng/ml				0.781					0.094
<8.1	860 (11.5)/7510	1.11 (1.05, 1.18)	0.001		759 (11.0)/6886	101 (16.2)/ 624	1.27 (1.00, 1.61)	0.050	
≥8.1	746 (10.3)/7233	1.13 (1.06, 1.20)	<0.001		670 (9.8)/6836	76 (19.1)/397	1.77 (1.33, 2.34)	<0.001	
Hcy, μmol/l				0.237					0.495
<10	305 (10.4)/2944	1.08 (1.00, 1.16)	0.042		271 (10.0)/2719	34 (15.1)/225	1.26 (0.83, 1.90)	0.274	
≥10	1310 (11.0)/11,914	1.15 (1.09, 1.21)	<0.001		1166 (10.5)/11110	144 (17.9)/804	1.53 (1.25, 1.87)	<0.001	
MTHFR C677T				0.119					0.579
CC	436 (10.7)/4092	1.09 (1.02, 1.17)	0.014		391 (10.2%)/3825	45 (16.9%)/267	1.39 (0.97, 1.99)	0.074	
CT	767 (10.5)/7312	1.17 (1.10, 1.25)	<0.001		677 (10.0%)/6799	90 (17.5%)/513	1.59 (1.22, 2.05)	0.001	
TT	412 (11.9)/3460	1.07 (0.97, 1.17)	0.170		369 (11.5%)/3208	43 (17.1%)/252	1.33 (0.92, 1.92)	0.127	

SBP = systolic blood pressure, DBP = diastolic blood pressure, TG = triglycerides, HDL = high-density lipoprotein, TC = total cholesterol, HCY = homocysteine, NOD = new-onset diabetes, MTHFR = methylenetetrahydrofolate reductase.

*Clinical center represents two rural areas in Anhui and Jiangsu provinces in China.

Adjusted for age, gender, clinical center, SBP, DBP, smoking and drinking status, baseline FPG, and BMI, if not stratified.

Only TG levels were analyzed because of colinearity with TC and HDL.

### Subgroup analyses of factors influencing the association between statins and NOD

We explored the role of other covariables on the association between statins and NOD. After removing the patients taking statins, Table 7 shows that basically the same associations were still observed.

**Table 7:** Odds ratios of lipid levels for NOD by logistic regression (remove the group of patients with statins).

Variables	Events (%)/n	Age-adjusted		Multivariate adjusted*	
		OR (95%CI)	P value	OR (95%CI)	P value
**TG**, **mmol/l**					
Continuous	1613 (10.9)/14,818	1.28 (1.22, 1.34)	<0.001	1.18 (1.13, 1.25)	<0.001
Categorical					
<1.7	899 (9.3)/ 9715	ref		ref	
>1.7	714 (14.0)/5103	1.60 (1.44, 1.77)	<0.001	1.35 (1.21, 1.51)	<0.001
**HDL**, **mmol/l**					
Continuous	1613 (10.9)/14,818	0.62 (0.53, 0.72)	<0.001	0.79 (0.67, 0.93)	0.005
Categorical					
<1.0 (males)/1.3 (females)	676 (12.8)/5276	ref		ref	
≥1.0 (males)/1.3 (females)	937 (9.8)/9542	0.74 (0.67, 0.82)	<0.001	0.82 (0.72, 0.92)	0.001
**TC**, **mmol/l**					
Continuous	1613 (10.9)/14,818	1.04 (0.99 1.09)	0.103	0.95 (0.90, 0.99)	0.030
Categorical					
<5.2	679 (10.8)/6313	ref		ref	
>5.2	934 (11.0)/8505	1.02 (0.92, 1.14)	0.662	0.87 (0.77, 0.97)	0.014
**TG/HDL**					
Continuous	1613 (10.9)/14,818	1.21 (1.17, 1.26)	<0.001	1.12 (1.08, 1.17)	<0.001
Categories					
<2.8	1435 (10.4)/13,789	ref		ref	
≥2.8	178 (17.3)/1029	1.80 (1.52, 2.14)	<0.001	1.46 (1.22, 1.75)	<0.001

Abbreviations: TG = triglycerides, HDL = high-density lipoprotein, TC = total cholesterol, NOD = new-onset diabetes.

*Adjusted for age, gender, clinical center, SBP, DBP, smoking and drinking status, baseline FPG, and BMI.

## Discussion

Previous studies showed that baseline lipid levels are important risk determinants of NOD among Caucasian populations^7–9^, but the association between lipid levels and NOD in Asian populations is unclear. Therefore, this study aimed to provide insights into the relationship between lipid levels and NOD in a Chinese hypertensive population. Results showed that TG and TG/HDL were independent risk factor for NOD in this Chinese hypertensive population. HDL had a protective effect for NOD.

The central pathophysiological feature in the development of type 2 diabetes mellitus from dyslipidemia is not clear. Despite the controversy, accumulating evidence indicates that both low HDL and high TG levels are early manifestations of insulin resistance (IR) and later diabetes, and that they can actively add to β-cell failure and participate in NOD onset^13^. In addition, HDL may also increase glucose disposal through direct effects in the skeletal muscle, the major site of glucose catabolism in the body^14^. Ginsberg *et al*.^15^ indicated that plasma TG may play a role in insulin resistance. Boden *et al*.^16^ suggested that elevated free fatty acids (FFA) may contribute to hyperglycemia by antagonizing the effects of insulin on endogenous glucose production and affecting insulin secretion. On the other hand, internal glycerol and fatty acids can be converted to glucose in the liver. A study revealed increased insulin levels in subjects with hypertension despite normal glucose levels. Nevertheless, the specific mechanisms linking lipids and NOD need to be further investigated.

In the present study, serum TG levels were a strong predictor of NOD in both genders, independent of the other risk factors. The association of fasting TG with NOD has been documented previously^8, 17, 18^, but these results were mainly reported using TG levels that were pooled with additional risk factors for diabetes or cardiovascular diseases^8^. Nevertheless, these previous studies support the present study, in which high TG levels significantly increased NOD risk by 18% if analyzed continuously or by 35% for high TG levels (≥1.7 mmol/l).

Low HDL is known to be an important predictor for the development of diabetes^7^, and certain agents known to raise HDL improve glucose metabolism and prevent diabetes^14^, but the protective effect among gender is still controversial. The present study confirmed that HDL protected against NOD. Continuous HDL levels decreased NOD risk by 21%, but without differences between genders, which suggests that the protective effect of HDL on NOD is not gender-specific in a Chinese hypertensive population.

The high TG and low HDL profile is the classical profile associated with the metabolic syndrome and other blood lipid abnormalities^19^, which have been associated with NOD^20^. Squillace *et al*.^21^ showed that high TG/HDL ratio increased the risk of NOD, independently of other traditional risk factors, supporting the results of the present study.

There are very few studies that observed the effect of TC on NOD. Mozaffarian *et al*.^22^. found that a lower total cholesterol to HDL cholesterol ratio (−4.7%; P < 0.001) was associated with a substantially lower incidence of diabetes. In the present study, TC had no effect on NOD when adjusted for age only, but after adjustment for multiple covariables, TC was negatively associated with NOD. This indicates that TC alone could have a protective effect against the development of NOD, but this effect is easily influenced by other factors. More studies are needed to confirm the relationship between TC and NOD.

Generally, physicians suggest lifestyle interventions to patients whose TG levels fall between 1.70 and 2.25 mmol/L, or lifestyle intervention combined with fibrate therapy to those whose TG levels falling between 2.26 and 4.5 mmol/L^23, 24^. From the present study, controlling the levels of TG for Chinese hypertensive people seems to be of great importance. TG levels can be easily changed by diet, therefore, diet and/or exercise may help to decrease the risk of NOD for hypertensive patients by decreasing TG levels. Studies are necessary to evaluate the impact of diet and exercise on NOD.

Statins are known to be associated with a higher risk of NOD^25–29^. In the present study, the sensitivity analysis showed that statins had no impact on NOD. These discrepancies could be due to the specific population being studied, including factors such as diet, lifestyle, and genetics. Additional studies are necessary to examine this point, but the impact of statins on adiponectin levels could be involved^30^.

There are potential limitations of our results. First, TC, HDL, and TG levels were only measured at baseline, thus the potential bias resulting from changes in TG and HDL over time cannot be ignored. Additionally, although laboratory parameters do not include measurements such as circulating insulin levels, they constitute a set of routine tests that are typically available to the practicing physician but not routinely used in screening. Thirdly, no adjustment could be done for changes in drugs during follow-up because of missing data. It has been shown that some classes of antihypertensive were associated with increased NOD risk^31^. Nevertheless, a review underlined that controlling the risk of NOD should not compromise blood pressure control^32^ and all subjects of the present study were taking angiotensin-converting enzyme inhibitors at baseline. Finally, diabetes onset can be affected by or result from diet and physical exercise, but we were not able to control for diet and exercise.

In conclusion, TG and TG/HDL were independent risk factors for NOD in this Chinese hypertensive population. HDL presented a protective effect for NOD. NOD was independent from TC.

## Material and Methods

### Study population

The subjects were from a randomized, double-blind, controlled trial (the CSPPT study)^33^ conducted from May 19, 2008, to August 24, 2013. This trial consisted of men and women aged 45–75 years old and with hypertension (defined as seated resting systolic blood pressure of >140 mmHg or diastolic blood pressure of >90 mmHg) at both the screening and recruitment visits, or who were taking at least one antihypertensive medication. A total of 20,702 people were included in the CSPPT and underwent a baseline examination in 2008. Fasting blood samples were collected for lipid analysis and genotyping of the MTHFR polymorphism. Eligible participants were randomly assigned in a 1:1 ratio to one of two treatment groups: a daily oral dose of one tablet containing 10 mg of enalapril and 0.8 mg of folic acid (the enalapril-folic acid group) or a daily oral dose of one tablet containing 10 mg of enalapril only (the enalapril-only group)^33^. The complete eligibility criteria and interventions are described in the CSPPT paper^33^.

**Figure 1:**
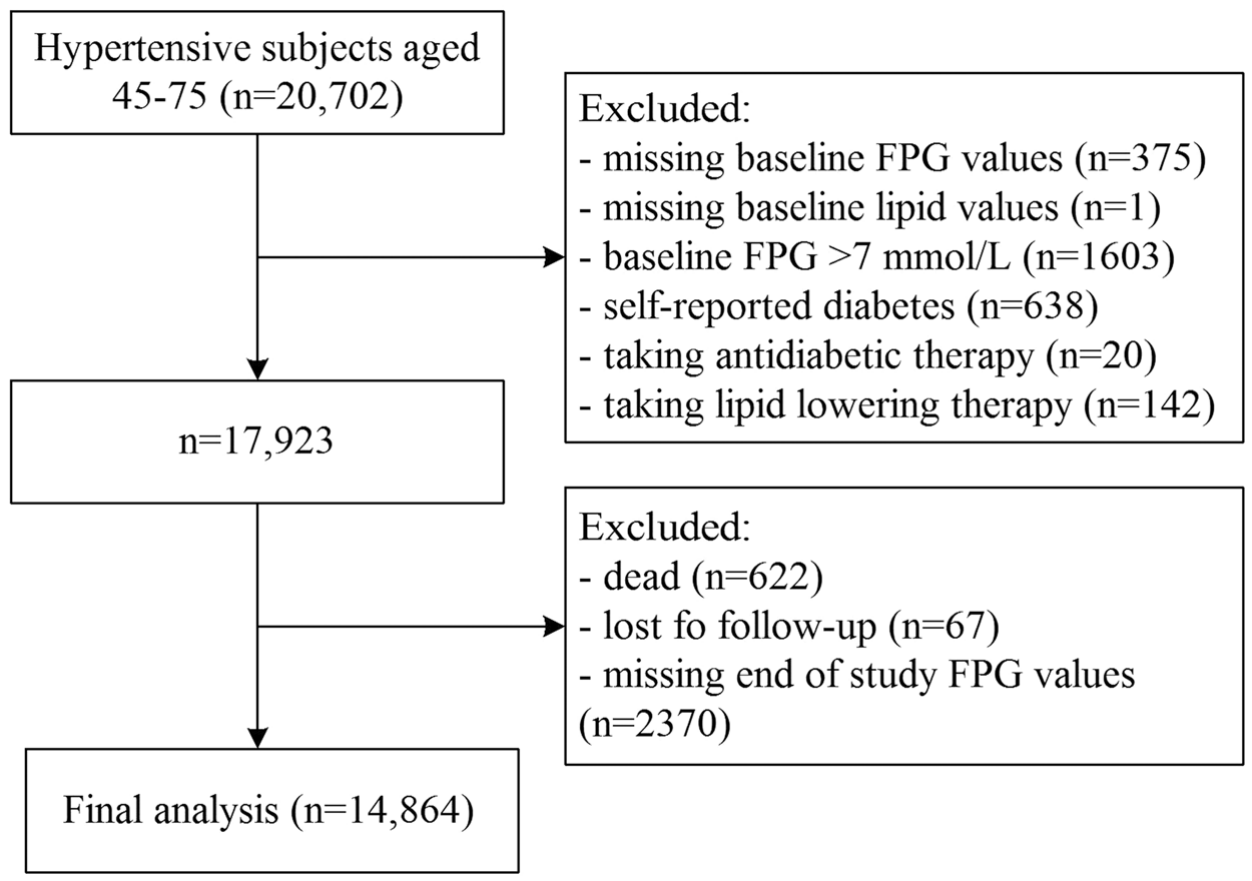
Subject flowchart.

The present post hoc study focused on those subjects with valid FPG values at baseline and at the end of study. Participants lacking lipid values were excluded. Those who had a self-reported diabetes history, patients whose FPG > 7 mmol/L at baseline or were taking hypoglycemic agents or lipid lowering therapy were also excluded. In addition, subjects with missing glucose values at the end of study, dead, or lost to follow-up were excluded as well. Thus, the present study included 14,864 non-diabetic subjects (6056 men and 8808 women) (Fig. 1).

The present study was approved by the ethics committee of the Nanfang Hospital, Guangzhou, China. The patients provided a written informed consent under the premises of the original CSPPT trial, including the possibility of post hoc analyses. This trial was registered with Clinicaltrials.gov (#NCT00794885). All methods were performed in accordance with the relevant guidelines and regulations.

### Data collection

Under the premises of the CSPPT trial^33^, all participants completed a detailed questionnaire assessing demographic, nutritional, lifestyle, and medical parameters. Height, weight, waist circumference, hip circumference, and blood pressure were recorded by trained medical staff. BMI was calculated as weight in kilograms (kg) divided by height in meters squared (m^2^). Serum Hcy, fasting TC, TG, HDL, and FPG levels at baseline and end of study were measured using automatic clinical analyzers (Beckman Coulter, Brea, CA, USA) at the National Clinical Research Center for Kidney Disease, Nanfang Hospital, Guangzhou, China. Smoking was recorded as never, former, or current. Alcohol drinking was recorded as never, former, and current.

NOD was defined as FPG ≥ 7.0 mmol/L at the end of study or self-reported physician diagnosis of diabetes or self-reported use of hypoglycemic agents during follow-up^34, 35^. The original CSPPT trial was conducted from May 19, 2008, to August 24, 2014. Follow-up was censored on August 24, 2014.

### Statistical analysis

All continuous data were evaluated for normality using plots. Normally distributed continuous variables were presented as mean ± standard deviation and analyzed using the Student t test. Non-normally distributed variables (Hcy and folate levels) were presented as median (interquartile range) and analyzed using the Kruskal-Wallis test. Categorical variables were presented as number and frequencies, and analyzed using the chi-square test. Logistic regression models were used to predict incident diabetes. The first model was adjusted for age only. The second model was adjusted for age and other confounders. Lipid-level variables were modeled as both continuous and binary: high TG levels and normal (≥1.7 vs. <1.7 mmol/L); low HDL levels and high [(<1.0 (males)/1.3 (females) vs. ≥1.0 (males)/1.3 (females)]; and normal TC levels and high (<5.2 vs. ≥5.2 mmol/L).

In the stratified analyses, the effects of lipid-level variables (both as continuous and binary) on the risks of NOD were estimated using logistic regression models among subgroups classified according to age (≥60 and <60), clinical center (Anqing and Lianyungang), gender (males and females), treatment group (enalapril and enalapril-folic acid), smoking status (never, ever, and current), alcohol (never, ever, and current), baseline BMI (<24, ≥24 and <28, and ≥28 kg/m^2^), SBP (<140, ≥140 and <160, ≥160 and <180, and ≥180 mmHg), DBP (<90, ≥90 and <100, ≥100 and <110, and ≥110 mmHg), folic acid (<8.1 and ≥8.1 ng/ml), Hcy (<10 and ≥10 μmol/l), and MTHFRC677T polymorphism (CC, CT, and TT). The results were presented as OR with 95%CI. An OR > 1 indicated an increased risk of NOD, while an OR < 1.0 indicates reduced risk. Possible confounding factors were taken into account, such as gender, clinical center, systolic and diastolic blood pressure, BMI, smoking, alcohol, and FPG. A double-tailed P-value < 0.05 was considered statistically significant in all analyses. Empowerstats (http://www.EmpowerStats.com.cn) and R (http://www.R-project.org/. version 3.2) were used to perform all statistical analyses.
